# On the Use of High‐Resolution and Deep‐Learning Seismic Catalogs for Short‐Term Earthquake Forecasts: Potential Benefits and Current Limitations

**DOI:** 10.1029/2022JB025202

**Published:** 2022-11-14

**Authors:** S. Mancini, M. Segou, M. J. Werner, T. Parsons, G. Beroza, L. Chiaraluce

**Affiliations:** ^1^ British Geological Survey Lyell Centre Edinburgh UK; ^2^ School of Earth Sciences University of Bristol Bristol UK; ^3^ Now at Scuola Superiore Meridionale Naples Italy; ^4^ United States Geological Survey Moffett Field CA USA; ^5^ Department of Geophysics Stanford University Stanford CA USA; ^6^ Istituto Nazionale di Geofisica e Vulcanologia Rome Italy

**Keywords:** short‐term earthquake forecast, high‐resolution seismic catalogs, Coulomb stress transfer, Epidemic‐Type Aftershock Sequence, operational earthquake forecasting

## Abstract

Enhanced earthquake catalogs provide detailed images of evolving seismic sequences. Currently, these data sets take some time to be released but will soon become available in real time. Here, we explore whether and how enhanced seismic catalogs feeding into established short‐term earthquake forecasting protocols may result in higher predictive skill. We consider three enhanced catalogs for the 2016–2017 Central Italy sequence, featuring a bulk completeness lower by at least two magnitude units compared to the real‐time catalog and an improved hypocentral resolution. We use them to inform a set of physical Coulomb Rate‐and‐State (CRS) and statistical Epidemic‐Type Aftershock Sequence (ETAS) models to forecast the space‐time occurrence of M3+ events during the first 6 months of the sequence. We track model performance using standard likelihood‐based metrics and compare their skill against the best‐performing CRS and ETAS models among those developed with the real‐time catalog. We find that while the incorporation of the triggering contributions from new small magnitude detections of the enhanced catalogs is beneficial for both types of forecasts, these models do not significantly outperform their respective near real‐time benchmarks. To explore the reasons behind this result, we perform targeted sensitivity tests that show how (a) the typical spatial discretizations of forecast experiments (≥2 km) hamper the ability of models to capture highly localized secondary triggering patterns and (b) differences in earthquake parameters (i.e., magnitude and hypocenters) reported in different catalogs can affect forecast evaluation. These findings will contribute toward improving forecast model design and evaluation strategies for next‐generation seismic catalogs.

## Key Points

- We compare retrospective forecast models informed by enhanced versus real‐time earthquake catalogs for the 2016–2017 Central Italy sequence
- To realize the benefits of high‐resolution catalogs, models should integrate advanced experimental setups, like finer spatial grids
- Results stimulate further testing on the optimal design of next‐generation forecast models based on enhanced seismic catalogs

## 1 Introduction

The last few years have seen the release of a rapidly increasing number of seismic catalogs developed by means of enhanced detection methods, including ones based on machine learning (e.g., Lapins et al., 2021; Liu et al., 2020; Ross et al., 2018). These advanced techniques reveal high‐resolution spatiotemporal characteristics of seismicity (e.g., Ross et al., 2019; Shelly, 2020; Tan, Waldhauser, Ellsworth, et al., 2021) that were previously untraceable in catalogs obtained through standard processing procedures (e.g., routine detections and analyst‐reviewed travel time measurements), whose real‐time implementation becomes particularly challenging during aftershock sequences. Seismologists have high hopes for this new generation of catalogs to open novel research avenues on unknown earthquake triggering mechanisms. Furthermore, since their compilation in real‐time conditions is now within reach (Zhu et al., 2022), enhanced seismic catalogs might ultimately boost the predictive skill of earthquake forecast models (Beroza et al., 2021). While the spatial resolution of current data sets is certainly impressive and allows describing in detail the cascading nature of earthquake occurrence (Arrowsmith et al., 2022) as well as fault geometries (Waldhauser et al., 2021a), how modelers should use the information encoded in those data products is not straightforward. For example, it is unclear how the high‐resolution details about the seismicity evolution can be adequately captured to improve probabilistic models that inform operational earthquake forecasts (Jordan et al., 2011). Over the last few years, seismic sequences such as those that occurred in the Central Apennines (Italy, 2016–2017) or in Ridgecrest (California, 2019) presented scientists the opportunity to develop and validate short‐term earthquake forecasts employing real‐time and near real‐time seismic catalogs and to calibrate modeling protocols for physical and statistical predictive models (Mancini et al., 2019, 2020; Marzocchi et al., 2017; Milner et al., 2020; Savran et al., 2020). Those experiments represented substantial advances in understanding how to improve the performance of physics‐based earthquake forecasts. This category includes continuum mechanics models that couple the coseismic Coulomb stress transfer among faults (Harris & Simpson, 1992) and laboratory‐derived friction laws describing the seismicity response on faults to these stress perturbations (e.g., Dieterich, 1994), commonly known as Coulomb Rate‐and‐State (CRS) forecasts. There are tantalizing indications from state‐of‐the‐art models that new information encoded in the seismicity patterns of next‐generation earthquake catalogs will lead to a step forward in their predictive skill. Recent experiments (Mancini et al., 2019, 2020) revealed that CRS models are potentially as informative as (and occasionally outperform) standard point‐process empirical models like the Epidemic‐Type Aftershock Sequence (ETAS; Ogata, 1988) models, but only when they incorporate high‐quality input data. These pseudo‐prospective (i.e., blind forward) tests concluded that stress triggering models particularly benefit from the inclusion of (a) optimized parameterization, (b) finite‐fault slip distributions, (c) small‐scale spatial variability of fault networks (receiver faults) with rupture kinematics informed from multiple data sources (e.g., past and unfolding focal mechanism solutions, smoothed regional stress inversions, mapped active faults, etc.), and (d) triggering contributions from smaller earthquakes (so‐called “secondary triggering”). The latter is a significant factor for models of earthquake interactions to track the fine‐scale evolution of the stress state that controls the local conditions for earthquake nucleation (Hanagan et al., 2022; Hanks, 1992; Helmstetter, 2003; Helmstetter et al., 2005; Marsan, 2005; Meier et al., 2014). Although counterevidence has been occasionally reported (e.g., Nandan et al., 2022), there is now a growing body of evidence supporting the notion that triggering contributions and local faulting patterns of small‐magnitude events help forecast larger earthquakes not only in stress‐based forecasts (Cattania et al., 2018; Mancini et al., 2019, 2020; Parsons et al., 2012; Segou & Parsons, 2014) but also for statistical models across long‐term time‐independent experiments (Helmstetter & Werner, 2012; Helmstetter et al., 2007; Werner et al., 2010, 2011) and short‐term time‐dependent tests (Helmstetter et al., 2006; Werner et al., 2011). Fully prospective evaluations by the Collaboratory for the Study of Earthquake Predictability (CSEP; Michael & Werner, 2018; Schorlemmer et al., 2018) corroborate these findings (Bayona et al., 2022; Zechar et al., 2013).

In this context, the jointly funded NERC‐NSFGEO project “*The Central Apennines Earthquake Cascade Under a New Microscope*” focused on the development of dramatically more comprehensive earthquake catalogs for the 2016–2017 Amatrice‐Visso‐Norcia (hereinafter, AVN) Italian seismic sequence using different techniques, which made it one of the best studied earthquake sequences ever. The AVN sequence started on 24 August 2016 at 01:36:32 UTC with an M6.0 event near Amatrice. During the following 5 months, earthquakes were triggered over a 60‐km long normal faulting region. Among these, the most notable were an M5.4 and an M5.9 at the northern termination of the activated fault system near the village of Visso, an M6.5 mainshock near Norcia, and four M5+ events in the Campotosto area (at the southernmost extent of the sequence).

In this work, we take advantage of the largest offline catalogs produced within the project, featuring a magnitude of completeness up to two units lower than the real‐time catalog, to develop the first set of CRS and ETAS short‐term earthquake forecasts based on high‐resolution and deep‐learning catalogs and tested in a retrospective experiment with the aim of (a) investigating if preliminary forecasts were already reliable enough or, conversely, we can provide higher predictability by informing current state‐of‐art modeling strategies with enhanced catalogs; (b) clarifying which key elements of these new catalogs (e.g., increased spatial clustering, event relocations, etc.) are the most beneficial, or detrimental, for models' performances; and (c) quantifying how the predictive skills of the forecasts improve when the assumed minimum triggering magnitude (*M*_MIN_) gradually decreases.

## 2 Data

We develop and test our forecasts using four out of the six catalogs released for the AVN sequence in the last 5 years. These catalogs present differences in the serial components of their development workflow starting from network geometries, detection, arrival time measurements, phase associations, locations, and magnitudes. We name them with progressive numbering, from CAT0 to CAT5. CAT0 is the real‐time catalog obtained by the Italian National Institute of Geophysics and Volcanology (INGV) monitoring system from data collected by the permanent Italian National Seismic Network (ISIDe Working Group, 2007); it includes 73,009 events from 24 August 2016 to 31 August 2017, reported with local magnitudes (MLs) and featuring a bulk completeness of *Mc* = 2.3 (Mancini et al., 2019). We do not employ the CAT1 and CAT2 catalogs as they are different realizations of the real‐time catalog, featuring the same CAT0 detections and magnitudes. The successively released catalogs all benefit from a much denser seismic network of 155 permanent and temporary stations deployed in the affected area following the M6.0 Amatrice earthquake (Moretti et al., 2016), and we categorize them as the “enhanced” catalogs. In the first, CAT3 (Michele et al., 2020; Spallarossa et al., 2020), detections are generated by an automated picker (Scafidi et al., 2018; Spallarossa et al., 2014) while the absolute hypocenters of its 440,727 events are obtained by a nonlinear location algorithm (Lomax et al., 2000). CAT3 also features an automated reevaluation of MLs and has a completeness of *Mc* = 0.6. Starting from the CAT3 locations, Waldhauser et al. (2021a) applied a double‐difference relocation algorithm with cross‐correlation‐based arrival time measurements to reduce the location error to only a few tens of meters, obtaining the high‐resolution CAT4 catalog, which comprises 390,336 events (with same MLs as CAT3) with *Mc* = 0.5. Finally, we use the deep‐learning‐derived CAT5 by Tan, Waldhauser, Ellsworth, et al. (2021), which is the largest catalog released so far for the sequence (*Mc* = 0.2), reporting 900,058 events with moment magnitudes until 15 August 2017. In CAT5, earthquakes are detected using the PhaseNet picker (Zhu & Beroza, 2019) based on a deep‐neural network, and relative locations are obtained by means of the hypoDD double‐difference method (Waldhauser & Ellsworth, 2000), but without the benefit of cross‐correlation‐based arrival time measurements. Since both CAT4 and CAT5 feature high‐precision relative relocations, we refer to them as the “high‐resolution” catalogs. For a more detailed description of the development process leading to each catalog and a thorough comparative illustration of the data sets used in this study, see Chiaraluce et al. (2022).

To calibrate the models' parameters, we use the same data as Mancini et al. (2019) to ensure consistency. They fit the rate‐and‐state and ETAS parameters on the M3+ presequence catalog (“learning phase catalog”) of the Italian Seismological Instrumental and Parametric Database (1990–2016 and 2005–2016 time periods for CRS and ETAS models, respectively) using the Maximum Likelihood Estimation approach by Zhuang et al. (2012). Likewise, our CRS models employ their set of finite‐fault slip models (FFMs; Chiaraluce et al., 2017; Scognamiglio et al., 2016; Tinti et al., 2016). To define the receiver‐fault matrix of the CRS models, we use their combination of kinematic parameters of large‐scale fault structures of the Central Apennines as described by the Database of Individual Seismogenic Sources (DISS Working Group, 2018) and focal mechanisms for the CRS learning phase reported in the Italian centroid moment tensor catalog.

## 3 Methods

CRS models presented in this study share a 6‐month forecast horizon (24 August 2016–24 February 2017) with the exception of the CRS model developed using CAT5 along with all ETAS models that are limited to the high‐rate period of the first 3 months of the sequence due to computational limitations. To ensure consistency and comparability of results, we adopt the same spatiotemporal model resolution of the benchmark forecasts previously published by Mancini et al. (2019). Thus, all forecasts (a) evolve through daily time windows and are updated either every 24 hr or at the occurrence of an M5.4+ earthquake and (b) are developed for a 2D gridded testing region of ∼150 km × 150 km centered on the M6.0 Amatrice earthquake and discretized with 0.02° (∼2 km) wide square bins. Coulomb stress change calculations for all CRS models are performed on cubic bins between 0 and 12 km of depth.

**Figure 1:**
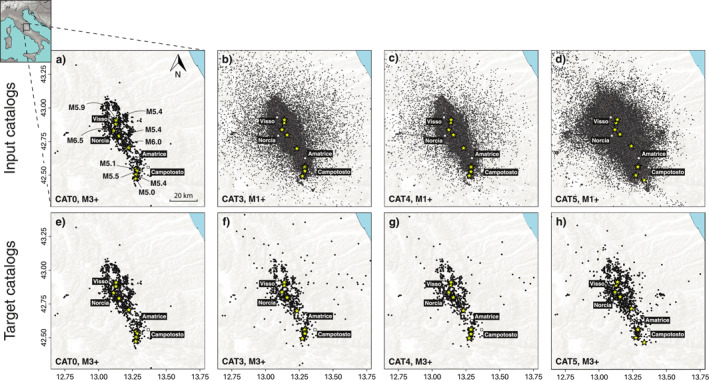
Input (a–d) and target (e–h) earthquake catalogs used in this study. Input catalogs inform the development of the models, and target catalogs are used to assess the performance of the forecasts. Yellow stars indicate the location of M5+ earthquakes.

To evaluate the added, if any, predictive value of forecasts that incorporate enhanced catalogs, we start by selecting the best performing near real‐time CRS and ETAS formulations by Mancini et al. (2019) as benchmarks. Here, we refer to them as CRS‐CAT0 and ETAS‐CAT0, respectively, where the second half of the name indicates the “input catalog” (or “input seismicity”) that informs the forecast model. The CRS‐CAT0 main modeling elements are as follows: (a) stress perturbations from M3+ events of the CAT0 input catalog (Figure 1a); (b) a heterogeneous background seismicity rate obtained by stochastic declustering (Zhuang et al., 2002) of the CRS learning phase catalog and smoothed in space with the adaptive kernel method by Helmstetter et al. (2007); (c) structural fault heterogeneities represented by a grid of spatially variable receiver planes derived by nearest neighbor association of (i) focal mechanisms included in the learning phase catalog (with random choice of nodal planes) and (ii) kinematic parameters from the DISS database in the off‐fault regions; (d) rate‐and‐state constitutive parameters fit on the CRS learning phase catalog (Aσ = 0.015 MPa and $\dot{\tau }$ = 1.9 · 10^−4^ MPa/year) and a fixed average coefficient of effective friction $\mu^{\prime }$ = 0.4; and (e) FFMs for the six M5.4+ events of the sequence: for all 3 ≤ *M* < 5.4 events devoid of an FFM, the model either (a) employs a synthetic fault with nodal plane randomly selected from their near real‐time moment tensor solution, empirically derived dimensions (Wells & Coppersmith, 1994) and uniform slip distribution from the moment relation of Hanks and Kanamori (1979) or (b) if no form of fault characterization is available, assumes an isotropic coseismic stress change distribution whose magnitude is directly proportional to the seismic moment and inversely proportional to the hypocentral distance (Chen et al., 2013).

Similarly, ETAS‐CAT0 projects seismicity generated by events with *M*_MIN_ = 3. Within each forecast window, this model considers the mean expected earthquake rates from 1,000 stochastic simulations of synthetic catalogs, with parameters fixed for the whole forecast horizon. Additional details about the formulations of the physical and statistical benchmarks, including the complete set of equations, are provided in Tables S1 and S2 in Supporting Information S1.

Developed with the same CRS‐CAT0 and ETAS‐CAT0 modeling principles and parameterizations, we generate six new forecast versions based on the newly available catalogs: CRS‐CAT3, ETAS‐CAT3, CRS‐CAT4, ETAS‐CAT4, CRS‐CAT5, and ETAS‐CAT5. Supported by the improvements in the completeness of the enhanced earthquake catalogs, we now extend the incorporation of secondary triggering over a wider range of minimum triggering magnitudes, down to M1 (Figures 1b–1d). Compared to the 1,067 M3+ stress sources used in CAT0 benchmark models, for a 6‐month experiment the potential number of stress‐perturbing (or “parent”) events increases by orders of magnitude: 52,159 and 49,862 for models developed with CAT3 and CAT4, respectively, and 148,211 events for the 3‐month CAT5 models.

We formally evaluate the performance of each forecast against the M3+ seismicity reported in the related catalog, that we name “target catalog” or “target seismicity” (Figures 1e–1h): for the 6‐month tests, 1,088 targets in CAT0, 835 in CAT3, and 809 CAT4. In CAT5 we consider the 998 targets of the first 3 months. We also cross‐validate models against the target seismicity of the enhanced catalogs assuming those are more representative of the observed earthquakes. We use standard metrics, such as the likelihood‐based S‐test (Zechar et al., 2010) to assess the absolute spatial consistency of the forecasts and the T‐test (information gain [IG] per earthquake; Rhoades et al., 2011) for the relative model ranking as established by the CSEP group. A description of the statistical metrics used in this paper is available in Supporting Information S1.

## 4 Results

In this section, we present the expected seismicity rate maps of forecasts based on different earthquake catalogs and compare them against the observations for specific periods during the AVN sequence. We then illustrate the statistical model performance evaluation, describing the absolute predictive power of the new models and their relative skill compared to the benchmark near real‐time forecasts.

### 4.1 Forecast Maps

**Figure 2:**
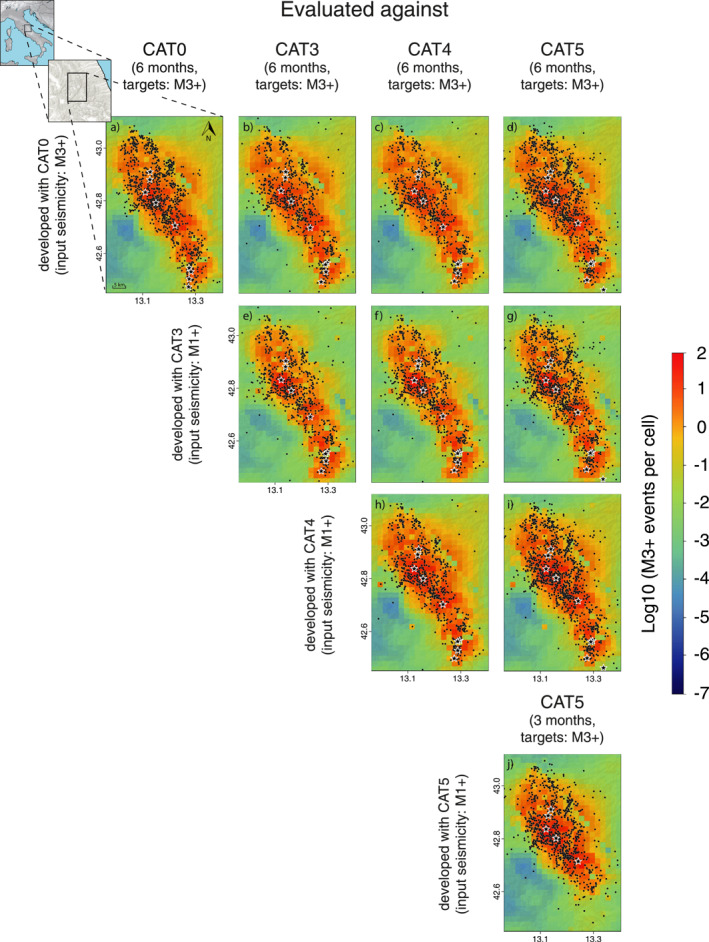
(a–j) Maps of expected seismicity rate for the Coulomb Rate‐State (CRS) models developed with and evaluated against the four catalogs. CAT0, CAT3, and CAT4 models cover a 6‐month forecast period, while CRS‐CAT5 has a 3‐month horizon. Each rate map is overlain with the corresponding target seismicity for the periods of interest: black stars for the M5+ earthquakes and black dots for the 3 ≤ *M* < 5 events.

In Figure 2 and Figure S1 in Supporting Information S1, we show all the forecast maps for physics‐based and statistical models, respectively, developed using the entire set of input catalogs, and we compare the expected rates against the M3+ observations as reported in each of the four catalogs. First, a preliminary visual analysis reveals that all forecasts satisfactorily bound the aftershock area, especially the stress‐based models (Figure 2). We observe only subtle differences between the near‐source seismicity rates projected by CRS and ETAS models developed with CAT0 and by the counterparts employing the enhanced catalogs. When we compare the CRS/ETAS‐CAT0 expected rates against the aftershock distributions reported in the other catalogs (Figures 2b–2d and Figures S1b–S1d in Supporting Information S1), we still find a good overall agreement, although some off‐fault target seismicity is not well captured, especially when we set the deep‐learning catalog (CAT5) as our target (Figure 2d and Figure S1d in Supporting Information S1). This off‐fault seismicity could be a result of misassociation, arrival‐time measurement error, or it could be real and a signature of delocalization of failure (Collettini et al., 2022). Conversely, CAT5 now reveals activity on branching faults to the north‐east of the main fault system, where the “CAT0” models did project heightened rates but no M3+ aftershocks were detected in real time.

CRS forecasts incorporating secondary triggering from M1+ events (Figures 2e–2j) explain some isolated target seismicity in the southwestern area of seismicity suppression (stress shadow) cast by the mainshocks (*e.g.*, CRS‐CAT4, Figures 2h and 2i). Nevertheless, some seismicity reported in the enhanced catalogs—but absent in CAT0—occasionally violates the shadow regions, indicating that further research is required to understand earthquake triggering in areas of coseismic stress reduction (e.g., Segou & Parsons, 2020). Within the set of new stress‐based models, we observe the main differences between the CRS‐CAT3 and CRS‐CAT4 models: (a) at the edges of the main aftershock zone, where the former features increased rates at the southern tip of the fault system and the latter presents higher rates in the rest of the peripheral area, and (b) at the near epicentral areas of the Visso and Norcia events where CRS‐CAT4 projects heightened seismicity. We attribute those findings to the relocation process, which is the only difference between the two catalogs. By contrast, the 3‐month CRS‐CAT5 does not present striking differences with respect to CRS‐CAT4 by visual inspection.

Similarly, there are only minor large‐scale visual differences between the preliminary ETAS (Figures S1a–S1d in Supporting Information S1) and the updated ETAS models implementing M1+ parent events (Figures S1e–S1j in Supporting Information S1); however, the near‐fault expected rates in ETAS‐CAT4 appear to be slightly lower than the other competing ETAS forecasts and more homogeneously distributed over a wider area. The possible reason behind this result is that CAT4 reports 35 M3+ parent earthquakes fewer than CAT3 (11 of which have *M* ≥ 4).

### 4.2 Likelihood Maps

**Figure 3:**
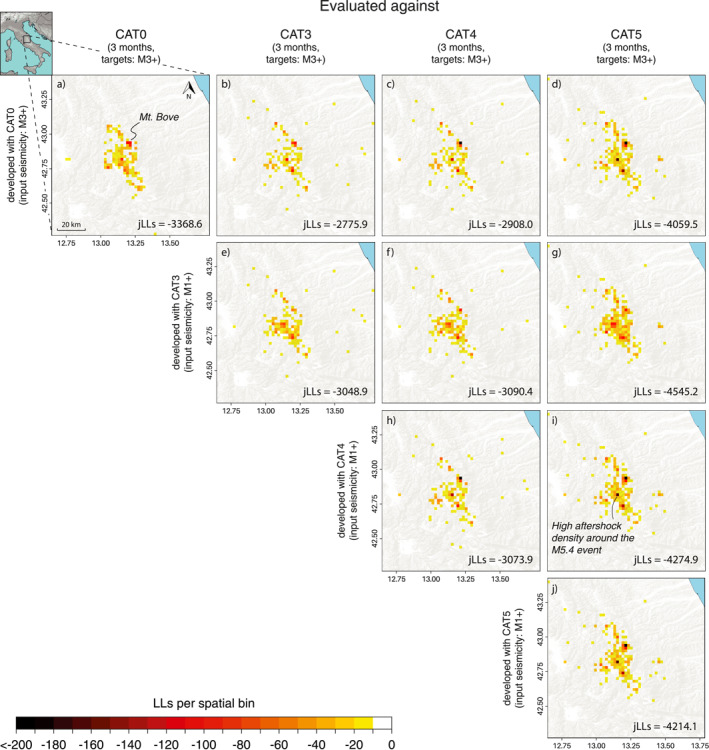
(a–j) S‐test's log‐likelihood (LL_S_) maps for the Coulomb Rate‐State models developed with and evaluated against the four generations of catalog. In each cell, LL_S_ values are aggregated over single daily forecast windows for a total period of 3 months. For each model, we report its joint log‐likelihood value when it is validated versus catalogs that are either equal or more evolved than the one used for its development.

In Figure 3 and Figure S2 in Supporting Information S1, we present the S‐test log‐likelihood (LL_S_) maps of the CRS and ETAS models, respectively, for the entire testing region aggregated over the daily forecast windows. Models with higher LL_S_ provide a better match with the observed earthquake locations. LL_S_ are calculated from normalized expected rates to isolate the forecasts' spatial performance. We first evaluate each model against its corresponding input catalog and then cross‐validate them against more evolved catalog generations. In general, we find that for all input‐target catalog combinations, the ETAS joint log‐likelihood (jLL_S_) values are higher than the CRS models, even when the latter are developed using the enhanced catalogs.

A persistent problem in the near real‐time CRS forecasts published by Mancini et al. (2019) was represented by the low LL_S_ values in the high clustering region around Mt. Bove (Figure 3a), which is the northern termination of the Mt. Vettore fault system activated by the M6.5 Norcia mainshock. We observe that model spatial performance in such critical regions, as well as in the wider near‐source area, improves when the physical model incorporates M1+ stress sources in CRS‐CAT3 (Figure 3e) but deteriorates again when models are evaluated against the relocated CAT4 and CAT5 catalogs (Figures 3f–3i). We also find that all CRS models evaluated using the enhanced catalogs (Figures 3b–3j) suffer from the presence of the now revealed, sparse off‐fault target seismicity which is instead missing in CAT0; this feature is particularly evident when models are validated against CAT5, which presents a previously undetected cluster of events to the east of the main aftershock zone. Surprisingly, the highest LL_S_ values are reached when the near real‐time model is evaluated versus the enhanced CAT3 and CAT4 catalogs (Figures 3b and 3c), suggesting that the overall spatial forecast of the CRS‐CAT0 model incorporating only the M3+ stress sources was already satisfactory. On the other hand, we obtain the lowest spatial consistency when models are validated against CAT5, presumably because of the presence of more newly detected target seismicity in off‐fault regions that alter the likelihood values at a very localized scale, especially when tightly clustered (Figure 3j).

The ETAS model ranking based on the LL_S_ values (Figure S2 in Supporting Information S1) follows that of the CRS models, with the ETAS‐CAT0 forecast evaluated against the CAT3 and CAT4 catalogs ranking among the best‐performing models. However, in this case ETAS‐CAT3 provides a slightly better spatial performance than the competing model versions.

### 4.3 Information Content of the Models

**Figure 4:**
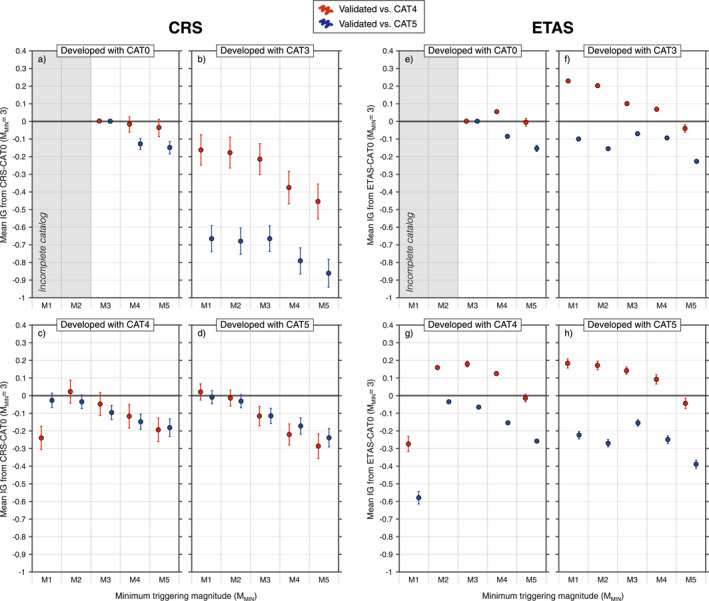
(a–d) Average daily information gain (IG) per earthquake from Coulomb Rate‐State (CRS)‐CAT0 of the whole set of CRS models for a cumulative 3‐month forecast horizon. Each of the CRS models developed with enhanced catalogs is presented in five versions implementing a different minimum triggering magnitude (*M*_MIN_) from M5 to M1. The *M*_MIN_ values for CRS‐CAT0 range from M5 to M3 due to the more limited completeness of the real‐time catalog (gray shaded area). A model is deemed more informative than the reference if its mean IG is positive and if its error bars do not cross the IG = 0 line. Red and blue symbols indicate models validated versus CAT4 and versus CAT5, respectively. Panels (e–h) same as the left panels but for the set of Epidemic‐Type Aftershock Sequence (ETAS) realizations.

For an overall assessment of the skill of the new model generations compared to the near real‐time forecasts, we calculate the IG per earthquake of each new CRS and ETAS model against the respective “CAT0” realization as a benchmark. We note that, unlike the LL_S_, IG scores are calculated from likelihoods derived from unnormalized seismic rates; therefore, this single metric accounts for model performance in reproducing both the spatial distribution of triggered earthquakes and their number. Positive IG values indicate a better performance of the model with respect to the selected benchmark, while negative values denote an information loss. To standardize the test and to facilitate the interpretation of the results, we (a) select a common 3‐month testing phase for all models and (b) employ a consistent testing catalog for each model‐benchmark couple (i.e., the likelihoods of model CRS/ETAS‐${CAT}_{i}$, with i = 0, 3, 4, 5, and of the benchmark CRS/ETAS‐CAT0 are both calculated against ${CAT}_{j}$, with j = 4, 5). Furthermore, to quantify the effect of incorporating gradually more complete input catalogs in the forecasting protocols, for each model, we illustrate how the IG scores vary when we consider different *M*_MIN_ thresholds (Figure 4).

At the same minimum triggering magnitude of the CRS‐CAT0 benchmark (*M*_MIN_ = 3) no CRS model has a statistically significant IG over the CRS‐CAT0 model developed with the monitoring room catalog (Figures 4a–4d). Instead, the performance of models developed using the enhanced catalogs oscillates from merely comparable (CRS‐CAT4, Figure 4c) to slightly poorer than CRS‐CAT0 (CRS‐CAT3 and CRS‐CAT5, Figures 4b and 4d). In any case, the information losses are no greater than 0.9 IG units (by comparison, Mancini et al. (2019) found that the CRS‐CAT0 model that we use here as benchmark reached IGs up to 8 units over simplistic CRS forecast models using real‐time data). When we extend the analysis to a wider range of minimum triggering magnitudes, from *M*_MIN_ = 1 to 5, we find that (a) within any single model IG values gradually rise as models incorporate secondary stress triggering from progressively smaller events and (b) no model is more informative than CRS‐CAT0. We observe this IG trend both when CRS models are validated versus CAT4 (red symbols in Figure 4) and versus CAT5 (blue symbols). This result confirms and emphasizes the importance of secondary triggering for the success of stress‐based models; however, for *M*_MIN_ ≤ 2 the IG tends to plateau (Figures 4a, 4b and 4d) and in some cases drops at *M*_MIN_ = 1 (CRS‐CAT4, Figure 4c). Therefore, the question arises of whether this outcome is due to the fact that *M* < 2 events do not contribute to the local M3+ aftershock triggering or instead reflects the limits of an insufficient model spatial resolution to describe stress patterns at a subkilometric level.

Another interesting aspect is the spread at any *M*_MIN_ threshold between the IG trends depicted by models evaluated against CAT4 and CAT5, as it is diagnostic of how the likelihood‐based model ranking is sensitive to catalog selection. We observe that while CRS models developed with CAT4/5 appear less sensitive to the choice of the target catalog, CRS‐CAT3 exhibits marked differences and performs worse when validated against CAT5 (∆IG≈ 0.5). This remark shows how alternate catalogs can describe the same sequence differently. The IG discrepancies between the CRS‐CAT3 and CRS‐CAT4 sets of models are surprising and beg the question on how stability of catalogs could be quantified during (or shortly after) their development.

We find that the IG trends of ETAS forecasts (Figures 4e–4h) mostly mirror those of physical models, confirming the general benefit from secondary triggering processes for statistical models as well. Similarly to the CRS counterparts, the ETAS IG values have the tendency to level out at *M*_MIN_ ≤ 2 (Figures 4f and 4h), and in the case of ETAS‐CAT4 they significantly fall at *M*_MIN_ = 1 (Figure 4g). Interestingly, ETAS realizations with *M*_MIN_ < 5 developed with the enhanced catalogs outperform ETAS‐CAT0 when they are evaluated against CAT4. On the other hand, they all rank slightly worse than the benchmark when CAT5 is set as testing catalog.

### 4.4 Sensitivity Tests

To explore the reasons behind our findings, we perform sensitivity tests on three potentially critical elements related to catalog development that could influence the performance of forecast models: the magnitude estimation, the event locations, and the spatial discretization.

**Figure 5:**
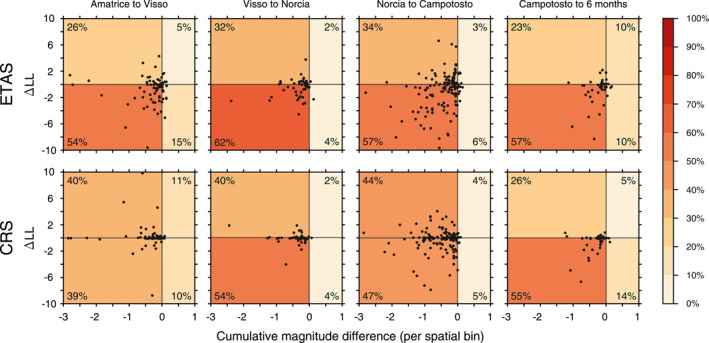
Cell‐wise differences in cumulative magnitudes between common source (parent) events of CAT0 and CAT4 versus resulting log‐likelihood (LL) differences in each spatial bin. LL values are obtained by using CAT4 target seismicity for both models. Colors follow the relative percentage ranges in the four quadrants.

According to the formulation of CRS and ETAS models, the higher the magnitude of a parent (or stress‐perturbing) event the larger the number of its directly triggered earthquakes and the area of influence of the spatial kernel over which they decay. In Figure 5, we plot the cumulative magnitude difference per spatial bin between the matching parent events of CAT4 (with automatically reestimated MLs) and CAT0 (with preliminary MLs) against the resulting cellwise LL differences when models developed using the two catalog generations are evaluated against CAT4. Overall, we find that the majority of those events present lower magnitudes in CAT4, although in most bins the cumulative differences are well below one unit of magnitude. For the ETAS model, we observe a rough visual agreement between information loss (i.e., a lower LL) and negative magnitude differences for ∼60% of cells throughout the duration of the sequence. Conversely, we find a more balanced ∆LL distribution for the CRS model, where several cells present an almost constant performance especially until the Norcia mainshock occurrence. Interestingly, for both models, we find that bins where the effect of magnitude re‐estimation appears most detrimental (up to 10 LL units after the Amatrice and Norcia mainshocks) are not those with the largest cumulative magnitude difference, underlining the importance of the employed spatial kernel.

**Figure 6:**
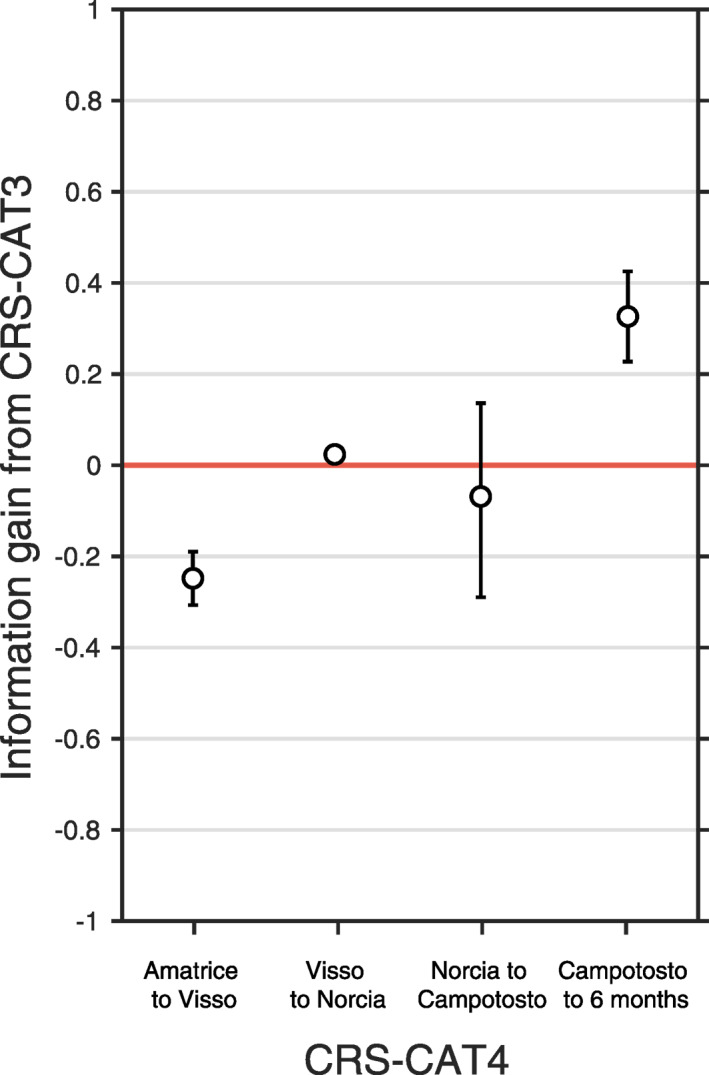
Average daily information gain per earthquake (IG) of Coulomb Rate‐State (CRS)‐CAT4 (developed with a relocated catalog) from CRS‐CAT3 (developed with a nonrelocated catalog). We plot the IG values for each period between the first four mainshocks and from the Campotosto events until 6‐months from the start of the sequence. CRS‐CAT4 is more informative than the reference at 95% confidence interval if IG values are positive and the error bars do not cross the red no‐gain line.

Relocated catalogs, such as CAT4, provide a highly clustered description of seismicity at local scales. Consequently, they aggregate seismicity in an increasingly smaller number of spatial bins and, in turn, increase the number of empty grid cells in the testing region. Their imprint on the spatial grid of the forecast perturbs the likelihood‐based scoring of the models due to differences in the spatial distribution of target seismicity. However, by visual inspection we see that the overall spatial differences arising from the relocation procedure in the clustering characteristics of CAT3 and CAT4 target seismicity are likely minimal at a regional scale (Figures 1f and 1g). Still, relocations of parent events may result in local redistribution of expected rates at the single spatial bin scale. To quantify such an effect, we compare between CRS‐CAT3 and CRS‐CAT4 (since CAT4 is the relocation of CAT3) when their performances are both evaluated against the relocated CAT4 catalog for time windows of interest in between mainshocks (Figure 6). As expected, the influence of relocation varies between high and low‐rate periods of the sequence, with results suggesting a weak information loss during the high‐rate period between the Amatrice and Visso earthquakes (IG ≈ −0.25) and a small IG at the low‐rate period after the Campotosto events (IG ≈ 0.35). Overall, the CRS‐CAT4 model does not present strong evidence for a better/worse performance. However, we note that the small differences between forecasts using non‐ and relocated earthquake catalogs could be attributed to the extremely large number of stations that recorded the sequence. Therefore, it could be argued that the effect of more precise hypocentral locations (i.e., with average relative horizontal location error <0.1 km in CAT4, about one order of magnitude smaller than in CAT3) is either negligible or not resolvable using our standard 2‐km spatial resolution.

**Figure 7:**
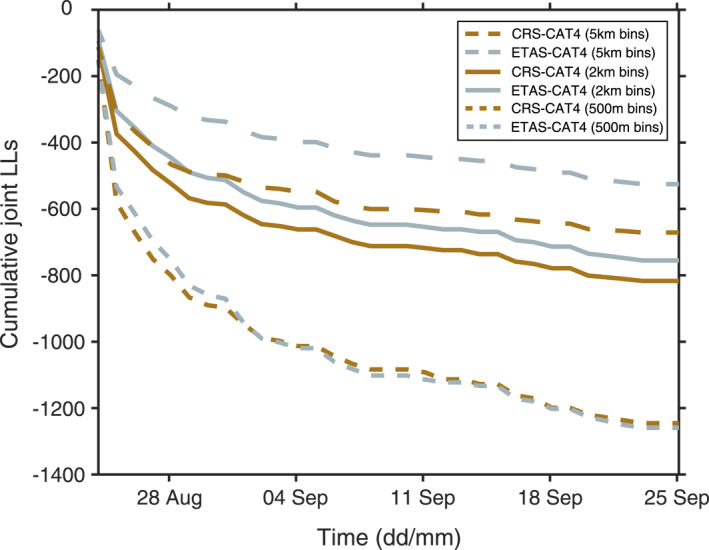
Cumulative spatial joint log‐likelihood (jLL_S_) during the first month of the sequence for the Coulomb Rate‐State (CRS)‐CAT4 and Epidemic‐Type Aftershock Sequence (ETAS)‐CAT4 models. We plot the spatial performance of the forecasts for three different spatial discretizations: 500 m, 2 km, and 5 km. The jLL_S_ trends are obtained by summing the S‐test log‐likelihoods of each bin and 1‐day time step.

The discretization of the testing region might hamper our ability to determine the effective local performance of models, particularly for forecasts based on enhanced catalogs that enable triggering contributions from very small magnitude events (e.g., source fault lengths are smaller than the grid spacing). In Figure 7, we illustrate the absolute performance of CRS‐CAT4 and ETAS‐CAT4 in terms of LL_S_ over time. We produce three versions of each model using a 5‐km, a 2‐km, and a 500‐m spatial binning for the first month of the sequence. Since more granular model resolutions imply lower probability of occurrence in any one cell, it is not surprising that absolute jLL values drop with finer model discretizations. Instead, here we focus on the relative $∆{LL}_{S}$ between CRS‐CAT4 and ETAS‐CAT4 within each binning category. We observe the maximum difference in likelihood between the two models at 5‐km binning ($∆{LL}_{S}$ = 150), which reduces by a half at 2‐km binning, and almost disappears at 500‐m discretization. These results suggest that stress‐based forecasts improve their spatial performance relative to ETAS when the stress field is resolved at a smaller scale, allowing a better description of the small fault segments (<1 km) contributing to the local evolution of the physical system. On the other hand, the spatial performance of ETAS‐like models, based on simulated catalogs instead of discrete point calculations, seems to be less affected by its resolution.

## 5 Discussion

In this study, the forecasts for the M3+ earthquakes of the 2016–2017 AVN sequence show no clear boost in their predictive skills when standard ETAS and best practice CRS modeling strategies are informed by enhanced, high‐resolution and deep‐learning earthquake catalogs. This supports the operational efficiency of the modeling strategy illustrated by Mancini et al. (2019, 2020) even when only real‐time data sets are employed.

We find that the new small‐magnitude events (1 ≤ *M* < 3) made available by enhanced catalogs have minor influence on the expected CRS and ETAS earthquake rates in the near source. This suggests that the near‐fault aftershock patterns may be largely dominated by the triggering effects from large to moderate events. On the other hand, we observe that the contribution of smaller earthquakes (a) can influence the off‐fault model validation and (b) is more likely effective at a finer scale. Accounting for the local triggering effects of these events improves the overall forecast performance, as shown by the trend of increasing IG when models implement gradually lower minimum triggering magnitudes (at least until *M*_MIN_ ≈ 2). This should encourage catalog developers to routinely produce more complete earthquake catalogs as seismic sequences unfold to allow testing future generations of forecasts in operational applications.

When we decrease the minimum triggering magnitudes to M1, however, we observe little to no additional IG, and in some cases even an information loss. From a modeling implementation perspective, as events approach such very small magnitudes their magnitude/location uncertainties and the radius of their triggering influence become comparable, which likely negates their ability to improve the forecast at the observational scales typically used. Nevertheless, this outcome also raises a more profound question for future model developments: is there a magnitude threshold below which physical fault‐to‐fault interactions become negligible or are our current modeling strategies approaching the limit of their predictability?

The sensitivity tests suggest that we cannot rule out either hypothesis, because (a) commonly adopted forecast spatial discretizations are inadequate to resolve localized triggering patterns revealed by high‐resolution catalogs, (b) seismic catalogs resulting from different workflows present remarkable differences even at moderate magnitudes (i.e., M3+) that might only be reflected in models by ad hoc parameter calibrations, and (c) the current likelihood‐based validation metrics are extremely susceptible to the choice of input and target seismicity and to the extent and resolution of the grid used to evaluate models. Regarding the latter point, it should be also considered that catalogs of tightly clustered seismicity clearly illustrate the existence of strong small‐scale space‐time dependencies among earthquakes that are not accounted for in standard forecast evaluation protocols established more than a decade ago (Schorlemmer et al., 2007). Those protocols assume independent Poisson distributions in each space‐time‐magnitude bin, responding to the earlier needs of testing longer term models in extended study areas (e.g., 5–20 years of M4.95+ seismicity in California). The rapid development of deep‐learning and other advanced techniques providing high‐resolution catalogs, refocuses scientists on the development of sequence‐specific earthquake forecasts evolving within shorter time frames (i.e., daily, or even hourly) over spatial extents of few tens of kilometers when seismicity is understandably non‐Poissonian in nature. Therefore, validation strategies will need to be redrawn free from these assumptions (Bayona et al., 2022; Savran et al., 2020) to adapt to the experimental setups made possible by modern enhanced data sets.

For the above reasons, a rigorous quantification of the added value of enhanced catalogs for short‐term earthquake forecasts is challenging at present, and model rankings presented here need to be interpreted with caution. We argue our standard binning (2 km) is the most limiting factor for properly resolving the triggering contributions of *M* < 2 earthquakes at a local (i.e., less than kilometric) scale. This is perhaps not too surprising as the source dimension for an M2 earthquake with nominal 3 MPa stress drop is ∼50 m, and it is supported by the fact that ETAS spatial consistency is superior to CRS at 5‐km binning, but with a 500‐m discretization the two models are equally informative. This is a promising result as it shows the potential for improving physics‐based model performance by resolving stress changes at a subcluster resolution of few hundred meters. A way forward will be to incorporate enhanced fault characterizations to capture the small‐scale variability of the receiver‐fault matrix. For example, motivated by the success of the implementation of time‐dependent receiver‐fault populations during the Ridgecrest sequence (Mancini et al., 2020), further work is needed to combine enhanced earthquake catalogs with information from richer rupture data sets (e.g., sets of focal mechanism solutions obtained with deep learning techniques or emerging fault structures progressively illuminated by high‐resolution catalogs). Moreover, future experiments on the adoption of enhanced catalogs for earthquake forecasting should consider testing 3D spatial models, potentially featuring locally variable or adaptive spatial discretization (Khawaja et al., 2022) over time (4D models). In this regard, current algorithms should become more computationally efficient, but the emerging cloud‐based capabilities for catalog development (e.g., QuakeFlow; Zhu et al., 2022) pave the way for real‐time applications.

Our results also show that forecasts developed with enhanced catalogs suffer from magnitude estimation resolution. The effect of magnitude inconsistencies on ETAS models' performance is not surprising as the magnitude of a parent event is directly related to the spatiotemporal distribution of triggered events. On the other hand, in physical models this translation is mediated through a series of operators (the slip distribution, the elastic dislocations, and the stress attenuation) that control the magnitude and spatial extent of the stress changes. We therefore stress the potential severe implications of magnitude inconsistencies in enhanced seismic catalogs (Herrmann & Marzocchi, 2020) on the performance of earthquake forecast with magnitude‐dependent productivity.

Although we find event relocations have a weak impact on IGs, we note that location uncertainties in relocated catalogs may perturb likelihood values, presumably at a cell‐wise level. This experiment does not provide sound evidence for a systematic influence of input seismicity relocations on models' predictive skills, but it is not uncommon for the relocation algorithms to deplete catalogs in very small magnitude events (e.g., CAT4 reports about 50,000 *M* < 2 earthquakes less compared to the nonrelocated CAT3), introducing obvious modifications in the input and target seismicity as well as nonlinear effects over space. Also, the set of AVN catalogs offer a striking example of how relocations of target seismicity might condition model performance evaluation. We refer to the M5.4 Campotosto event, whose epicentral location among the four catalogs spreads over about 4 km (Figure 1). This is likely due to the availability of either real‐time, near real‐time or offline data to constrain the location and should warn modelers on how variable input and target seismic patterns return a different model score. The fact that model ranking could be significantly influenced by the presence/absence of very few events in a small number of isolated cells (e.g., the cluster of earthquakes newly detected by CAT5 at the eastern off‐fault region) underlines the necessity for more objectively defined testing regions in earthquake forecasting experiments.

A major obstacle for an unbiased estimate of the predictive skill of short‐term forecast models informed by enhanced catalogs is that we cannot know a priori which catalog, if any, more closely represents the ground truth.

## 6 Conclusions

This study set out to explore the potential for improving physics‐based and statistical short‐term earthquake forecasts for the 2016–2017 Central Italy seismic cascade by incorporating additional information provided by enhanced, high‐resolution and deep‐learning earthquake catalogs released a few years after the sequence. To that end, we design a retrospective experiment where the best‐performing CRS and ETAS forecasts among those generated by Mancini et al. (2019) using near real‐time data are set as benchmarks to measure any performance improvement of the updated models. Given their much lower magnitude of completeness (*Mc* < 1), the enhanced catalogs enable the incorporation of the secondary triggering contributions from all M1+ earthquakes to forecast the space‐time occurrence of the M3+ events.

From the absolute and comparative evaluation of models' predictive skills, based on the typically used S‐ and T‐tests, we observe that most forecasts developed with the enhanced catalogs are not more informative than those based on the preliminary monitoring room catalog. We observe, however, that in the set of new CRS and ETAS models the predictive skills improve with decreasing minimum triggering magnitude from M5 to M2, while fixing *M*_MIN_ = 1 appears ineffective and occasionally even detrimental for model performance within the same 2‐km grid.

Despite its exploratory nature, this study offers valuable insights into the issues that modelers are likely to face soon. Notwithstanding the fact that current enhanced seismic catalogs provide an unprecedented quality description of earthquake occurrence, each of them illustrates a different version of it. These catalogs are products of different choices in their serial components of detection, event association, and seismic parameters estimation that make it difficult to quantify the contribution of each choice toward improving earthquake forecasts. As catalog development in the era of deep‐learning becomes more widespread, we will likely see more target‐specific catalogs in place holding increased testing capability against benchmark data sets.

Furthermore, likelihood‐based scores are extremely sensitive to such uncertainties, and the most commonly used model spatial discretizations (from the standard 10 km of CSEP tests to the 2 km of the present experiment) are likely inadequate to evaluate highly localized triggering patterns of clustered, non‐Poissonian seismicity. This experiment also provides a step forward in understanding the relevance of secondary triggering interactions in a high clustering environment, but more work is needed to characterize the event‐specific triggering potential to probe subcluster triggering mechanisms. In this regard, the fact that resolving the static stress variability at a finer scale is beneficial for CRS models should encourage catalog developers to produce and release enhanced catalogs in real time. We believe that deep‐learning‐based forecasts shared with the community will promote detailed investigations in the wake of those presented in this study and motivate further research on model validation strategies.

To conclude, our findings illustrate some of the limitations of currently popular modeling protocols and evaluation metrics but are also an invitation to promote further probing on the actual power of enhanced catalogs for earthquake forecasting.

## Erratum

In the originally published version of this article, the following sentence has been added in the Acknowledgments. “Any use of trade, firm, or product names is for descriptive purposes only and does not imply endorsement by the U.S. Government.”. This may be considered the authoritative version of record.

## Supporting information

Supporting Information S1

## Data Availability

The seismic catalogs employed in this paper are currently available at the individual repositories indicated in their related publications. The real‐time catalog (here named CAT0) by the INGV seismic monitoring room (ISIDe Working Group, 2007) can be searched at http://cnt.rm.ingv.it/en. The CAT3 catalog by Spallarossa et al. (2020) can be acquired at the following Zenodo repository: https://doi.org/10.5281/zenodo.4306165 (Michele et al., 2020). CAT4 (Waldhauser et al., 2021a) is available at https://doi.org/10.5281/zenodo.5091137 (Waldhauser et al., 2021b). CAT5 catalog by Tan, Waldhauser, Ellsworth, et al. (2021) is accessible at the Zenodo repository https://doi.org/10.5281/zenodo.4736089 (Tan, Waldhauser, & Ellsworth, 2021). A comprehensive repository for all the catalogs developed for the AVN sequence (including CAT1 and CAT2, not employed in this paper) is available at http://doi.org/10.5285/5afccfe5-142e-4e93-a6cc-55216fa1db06. The near real‐time moment tensor solutions have been taken from https://doi.org/10.13127/TDMT (Scognamiglio et al., 2006). The Italian centroid moment tensor data set is available at https://doi.org/10.13127/rcmt/italy (Pondrelli & Salimbeni, 2006). The Database of Individual Seismogenic Sources for Italy and surrounding areas (version 3.2.1) is downloadable at https://doi.org/10.13127/diss3.3.0 (DISS Working Group, 2018). The calculations for the Coulomb Rate‐and‐State forecasts were performed using the code “CRS” by Cattania and Khalid (2016), available at https://github.com/camcat/crs. Maps presented in this paper have been produced using QGIS (http://www.qgis.org).
