# Supplementary material for: On the Use of High‐Resolution and Deep‐Learning Seismic Catalogs for Short‐Term Earthquake Forecasts: Potential Benefits and Current Limitations
Source: J Geophys Res Solid Earth. 2022 Nov 14;127(11):e2022JB025202. doi: 10.1029/2022JB025202 (PMC9787792; doi:10.1029/2022JB025202)
Supplement: Supplementary file 1 — Supporting Information S1 [file JGRB-127-e2022JB025202-s001.pdf]

Journal of Geophysical Research

Supporting Information for

On the Use of High-Resolution and Deep-Learning Seismic Catalogs for Short-Term Earthquake Forecasts: Potential Benefits and Current Limitations

S. Mancini [1,2,3], M. Segou[1], M. J. Werner[2], T. Parsons[4], G. Beroza[5], and L. Chiaraluce[6]

[1] British Geological Survey, Lyell Centre, Edinburgh, UK

[2] School of Earth Sciences, University of Bristol, Bristol, UK

[3] *now at*: Scuola Superiore Meridionale, Naples, Italy

[4] United States Geological Survey, Moffett Field, CA, USA

[5] Department of Geophysics, Stanford University, Stanford, CA, USA

[6] Istituto Nazionale di Geofisica e Vulcanologia, Rome, Italy

Contents of this file

Text S1 to S3
Figures S1 and S2
Tables S1 and S2

Introduction

In this supplement we present the basic mathematical framework behind the CRS (text S1) and ETAS (text S2) models presented in the main text. We furthermore illustrate the statistical formulation of the two CSEP tests that we employed to evaluate the performance of the forecasts (text S3).

We also include additional maps for the ETAS models. Figure S1 represents the ETAS expected rates for model versions developed with and tested against the four catalogs of the AVN sequence. Figure S2 shows S-test's log-likelihood (LL_S) maps of models presented in Figure S1.

Text S1. Coulomb Rate-State modeling framework

The Coulomb stress calculations presented in this study assume an elastic medium (*Okada*, 1992), with average values of shear modulus and Poisson's ratio for the upper crust (30 GPa and ν=0.25, respectively). Static Coulomb stress changes are formally defined by *Rice* (1992);

$$\Delta CFF = \Delta\tau + \mu'(\Delta\sigma), \qquad (S1)$$

with $\Delta\tau$ the shear stress perturbation resolved on an established receiver fault orientation (positive in direction of fault slip), $\Delta\sigma$ the normal stress change, $\mu' = \mu(1 - B)$ the effective coefficient of friction (where B is the Skempton's coefficient accounting for pore pressure changes resulting from a stress application).

To account for the time dependency of seismicity, the expected earthquake nucleation rates in response to the CFF perturbation are calculated by means of the rate-and-state friction constitutive laws. We consider the standard formulation by *Dieterich* (1994) (hereinafter, D94), where the space-time earthquake rate (R) evolves as:

$$R(t, x, y) = \frac{r_0(x, y)}{\gamma(t)\dot{\tau}}, \qquad (S2)$$

where r_0 is the background seismicity rate, $\dot{\tau}$ the secular shear stressing rate and γ a state variable. When stress conditions are stable, the latter has a value given by:

$$\gamma_0 = \frac{1}{\dot{\tau}}. \quad \text{(S3)}$$

However, when a stress perturbation is imparted on a receiver fault, its γ instantaneously changes as:

$$\gamma_n = \gamma_{n-1} \exp\left(\frac{-\Delta S}{A\sigma}\right), \quad \text{(S4)}$$

where ΔS and $A\sigma$ represent the shear stress change from the source event and the normal component of the stress on the fault plane, respectively. γ_n and γ_{n-1} in equation (S4) indicate the state of the system before and after the stress perturbation. To use the Coulomb stress change in the CRS framework instead of the sole shear stress change, *Dieterich et al.* (2000) incorporated the contribution of the normal stress change as well through a positive non-dimensional parameter (α), setting:

$$S = \tau - (\mu - \alpha)(1 - B)\sigma. \quad \text{(S5)}$$

By comparing equations (S1) and (S5), it follows that S is approximated to the CFF by assuming that $\mu' = (\mu - \alpha)(1 - B)$.
The state variable evolves as:

$$d\gamma = \frac{1}{A\sigma}[dt - \gamma dS]. \quad \text{(S6)}$$

When a receiver fault experiences a positive stress change its γ value decreases, therefore it is expected to produce a larger number of triggered earthquakes. If no further stress step is imposed, the seismic rate recovers as the state variable evolves at each time step (Δt) as:

$$\gamma_{n+1} = \left(\gamma_n - \frac{1}{\dot{\tau}}\right) \exp\left(\frac{-\Delta t \dot{\tau}}{A\sigma}\right) + \frac{1}{\dot{\tau}}\ , \quad \text{(S7)}$$

Furthermore, in the D94 formulation the time required for the seismic rate (R) to return to the background level (r_0) is given by:

$$\boldsymbol{t_a} = \frac{\boldsymbol{A\sigma}}{\dot{\boldsymbol{\tau}}}. \qquad (\mathbf{S8})$$

Text S2. The ETAS model

ETAS (*Ogata*, 1988) is a stochastic point process model of seismicity that evolves according to a branching pattern. The main characteristics of ETAS are: (1) each earthquake triggers its offspring events which can potentially have larger magnitude than their parent, (2) the abundance of triggered events depends on the magnitude of the parent, and (3) the number of triggered earthquakes decays in time according to the Omori law. The expected seismicity rate in space (x,y) and time (t) is called "conditional intensity" (λ) and is given by the summation of the background rate μ (a time-independent and spatially heterogeneous Poisson process) plus a triggering function accounting for the triggering history (H_t) from all previous earthquakes occurred at $t_i < t$:

$$\lambda(x, y, t \mid H_t) = \mu(x, y) + \sum_{i:t_i<t} g(t - t_i, x - x_i, y - y_i; M_i). \qquad (S9)$$

Ogata (1998) illustrates that the triggering function is composed of empirical relations:

$$g(t, x, y; M) = K_0 e^{\alpha(M - M_{cut})} \cdot c^{p-1}(t + c)^{-p}(p - 1) \cdot f(x, y|M). \qquad (S10)$$

The aftershock productivity by a parent event with magnitude $M \leq M_{cut}$ is regulated by the K_0 parameter, while the α parameter sets the efficiency of parent events in triggering aftershocks as a function of magnitude. The c and p parameters belong to the modified Omori law and describe the distribution of triggered events in time. The term $f(x, y|M)$ describes the probability distribution of the aftershocks' location and of their spatial decay around the triggering event given the parent's magnitude. We use an isotropic power law distribution defined as:

$$f(x, y|M) = \left(d\, e^{\gamma(M - M_{cut})}\right)^{q-1} / \pi \cdot \left(x^2 + y^2 + d \cdot e^{\gamma(M_i - M_{cut})}\right)^{-q} (q - 1), \qquad (S11)$$

where d, q and γ concur toward scaling the radii of influence of parent earthquakes depending on their magnitude.

Text S3. Performance evaluation metrics

Among the tests employed withing the Collaboratory for the Study of Earthquake Predictability (CSEP), we use here (1) the S-test's log-likelihood scores to measure the spatial performance of the forecasts, and (2) the T-test for a comparative evaluation of the relative model performance by means of the information gain per earthquake (IG) metric.
For each spatial cell of the target region, the log-likelihood (LL) of observing ω events given a forecast of λ earthquakes is defined as the logarithm of the probability $Pr(\omega|\lambda)$ (*Schorlemmer et al.*, 2007):

$$LL(\omega|\lambda) = \log\big(Pr(\omega|\lambda)\big) = -\lambda + \omega log\lambda - \log(\omega!). \quad \text{(S12)}$$

The joint log-likelihood is obtained by summing over all the cells (*i,j*) of the region:

$$jLL(\Omega|\Lambda) = \sum_{(i,j)\in R} \Big(-\lambda(i,j) + \omega(i,j)\, log\big(\lambda(i,j)\big) - log(\omega(i,j)!)\Big), \quad \text{(S13)}$$

with Ω and Λ the observed and modeled catalogs, respectively.
By definition, LL values are negative. Therefore, the closer LL values are to zero the better is the match between model and the observed earthquakes. In the S-test (*Zechar et al.*, 2010), log-likelihoods are calculated from normalized expected earthquake rates, so that the spatial component of the forecast is isolated from the temporal component. To do this, we assume that the total number of expected events equals the total number of observed events.

The T-test is used to assess the relative performance of a model compared to a benchmark (*Rhoades et al.*, 2011). This test ranks forecasts according to the metric of the information gain per earthquake (IG), which is defined as the average difference in log-likelihood per earthquake between a model A and a benchmark B:

$$IG(A,B) = \frac{jLL_A - jLL_B}{N}, \tag{S14}$$

where N is the total number of observed earthquakes.

Please note that, to account for model performance in reproducing both the spatial distribution of triggered earthquakes and their abundancy, the T-test uses log-likelihoods calculated from unnormalized expected earthquake rates. To calculate the 95% confidence interval around this average difference, we use the procedure by *Rhoades et al.* (2011), fixing a significance level α = 95%. If IG = 0, the model and its benchmark have the same performance compared to the observations.

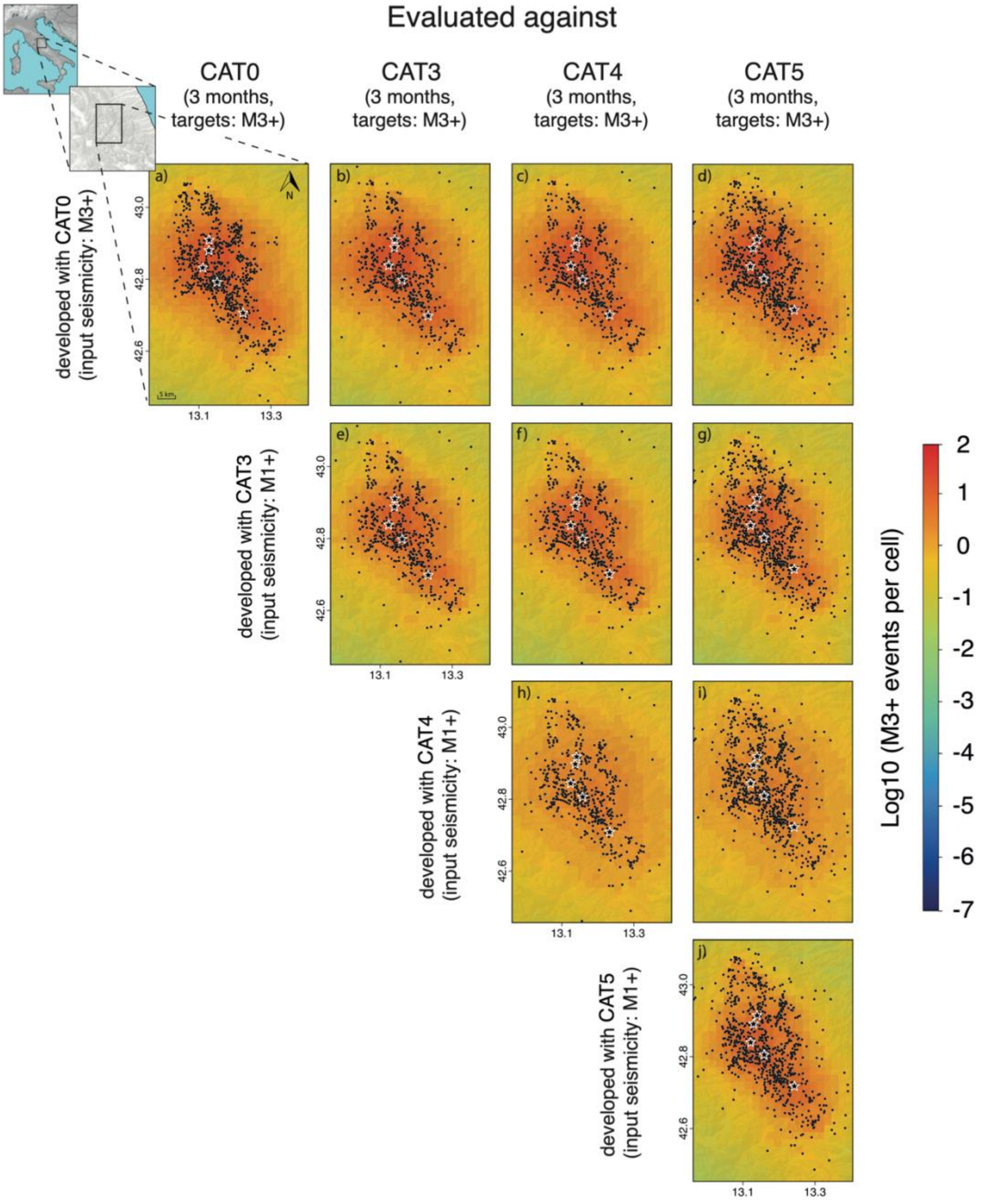

Figure S1. Maps of expected seismicity rate for the Epidemic-Type Aftershock Sequence (ETAS) models developed with and evaluated against the four catalog generations for a 3-month period. Each rate map is overlaid with the corresponding target seismicity for the periods of interest: black stars for the M5+ earthquakes and black dots for the 3≤M<5 events.

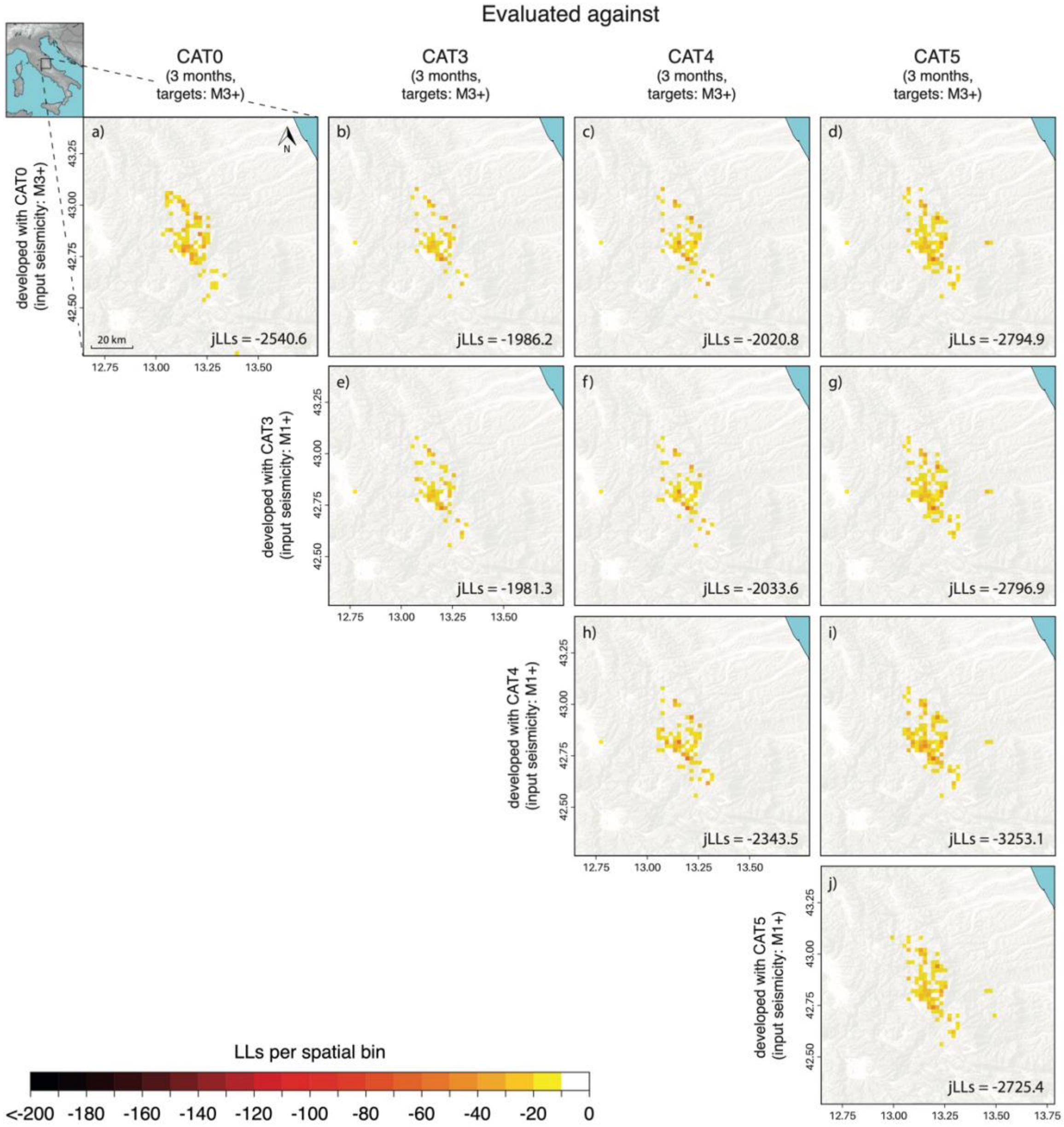

Figure S2. S-test's log-likelihood maps for the ETAS models developed with and evaluated against the four catalog generations for a 3-month period. For each model, we report its joint log-likelihood value when it is validated vs. catalogs that are either equal or more evolved than the one used for its development.

Table S1. Parameters used for the all ETAS simulations, with Mcut = 3.0 and α = β = b log(10)

Parameters	Value	Description
K_0	0.095 ± 0.009	Productivity parameter
c (days)	0.013 ± 0.005	Omori-Utsu c-value
p	1.184 ± 0.05	Omori-Utsu p-value
d (km^2)	1.686 ± 0.31	Spatial kernel parameter
q	2.757 ± 0.20	Spatial kernel parameter
γ	1.183 ± 0.058	Aftershock zone scaling factor
Bg-rate (events/day)	0.035	ETAS background rate (M ≥ 3.0)
n	0.97	Branching ratio

Table S2. Main features of CRS models. Mmin = minimum magnitude for stress sources; USD = uniform slip distribution; FFM = finite-fault rupture model; I = isotropic stress field; SUP = spatially uniform receiver planes; SVP = spatially variable planes.

Model name	Input catalog	Stress Calculations					Rate-and-State Parameters (Optimized on CRS learning catalog)		
		Secondary Triggering	M_{min}	Slip Distribution	μ'	Receiver faults	r_0	$A\sigma$ (MPa)	$\dot{\tau}$ (MPa/yr)
CRS-CAT0	CAT0	Yes	3.0	FFM (M ≥ 5.4)<br>USD (M ≥ 4.0)<br>I (M ≥ 3.0)	0.4	SVP	Spatially Heterogeneous	0.015	0.00019
CRS-CAT3	CAT3	Yes	1.0	FFM (M ≥ 5.4)<br>USD (M ≥ 4.0)<br>I (M ≥ 1.0)	0.4	SVP	Spatially Heterogeneous	0.015	0.00019
CRS-CAT4	CAT4	Yes	1.0	FFM (M ≥ 5.4)<br>USD (M ≥ 4.0)<br>I (M ≥ 1.0)	0.4	SVP	Spatially Heterogeneous	0.015	0.00019
CRS-CAT5	CAT5	Yes	1.0	FFM (M ≥ 5.4)<br>USD (M ≥ 4.0)<br>I (M ≥ 1.0)	0.4	SVP	Spatially Heterogeneous	0.015	0.00019
